# Rational design of ultrastable and reversibly photoswitchable fluorescent proteins for super-resolution imaging of the bacterial periplasm

**DOI:** 10.1038/srep18459

**Published:** 2016-01-06

**Authors:** Mariam El Khatib, Alexandre Martins, Dominique Bourgeois, Jacques-Philippe Colletier, Virgile Adam

**Affiliations:** 1 Univ. Grenoble Alpes, IBS, F-38044 Grenoble, France; 2 CNRS, IBS, F-38044 Grenoble, France; 3 CEA, IBS, F-38044 Grenoble, France

## Abstract

Phototransformable fluorescent proteins are central to several nanoscopy approaches. As yet however, there is no available variant allowing super-resolution imaging in cell compartments that maintain oxidative conditions. Here, we report the rational design of two reversibly switchable fluorescent proteins able to fold and photoswitch in the bacterial periplasm, rsFolder and rsFolder2. rsFolder was designed by hybridisation of Superfolder-GFP with rsEGFP2, and inherited the fast folding properties of the former together with the rapid switching of the latter, but at the cost of a reduced switching contrast. Structural characterisation of the switching mechanisms of rsFolder and rsEGFP2 revealed different scenarios for chromophore *cis*-*trans* isomerisation and allowed designing rsFolder2, a variant of rsFolder that exhibits improved switching contrast and is amenable to RESOLFT nanoscopy. The rsFolders can be efficiently expressed in the *E. coli* periplasm, opening the door to the nanoscale investigation of proteins localised in hitherto non-observable cellular compartments.

The nanoscale visualisation of intracellular details in live cells by super-resolution microscopy often relies on employing “phototransformable” fluorescent proteins (PTFPs) as genetically encoded markers^1^. Accordingly, the engineering of PTFPs with improved biochemical or photophysical properties has fostered the development of a large variety of nanoscopy approaches^2^,^3^. Notably, methods such as RESOLFT (REversible Saturable OpticaL Fluorescence Transitions)^4^, nonlinear SIM (Structured Illumination Microscopy)^5^ or pcSOFI (photochromic Stochastic Optical Fluctuation Imaging)^6^ exploit reversibly switchable fluorescent proteins (RSFPs) that are able to repeatedly toggle between a fluorescent (*‘on’*) and a non-fluorescent (*‘off’*) state. In so-called “negative switching” green RSFPs, the *on*-to-*off* transition competes with fluorescence emission upon illumination by cyan light (~490 nm), while the *off*-to-*on* transition promptly responds to illumination by violet light (~405 nm). The negative RSFP’s subfamily first consisted of Dronpa^7^ and its variants^8^,^9^, and was progressively enriched with other proteins of anthozoan origin (corals and anemones) such as rsCherryRev^10^, rsTagRFP^11^, the mGeos’s^12^ and the biphotochromic IrisFP^13^ and NijiFP^14^. However, the development of RESOLFT nanoscopy in live cells necessitates RSFPs that switch efficiently even at low illumination power (to minimise photo-damage), display minimal residual fluorescence in the *off* state (to maximise contrast), and are highly resistant against switching fatigue (to sustain a large number of successive *on*-*off* switching cycles). It was found that variants engineered from fluorescent proteins of hydrozoan origin (jellyfishes), and notably from the well-known EGFP, could fulfil these requirements, giving rise to rsEGFP^15^ and rsEGFP2^16^. Very recently, variants of rsEGFP obtained by directed evolution were reported that mature and express more efficiently in the cytosol of mammalian cells^17^. RSFPs of anthozoan origin with enhanced photoswitching properties were also introduced, including the positive switcher Kohinoor^18^ evolved from Padron^8^, and the negative switcher Skylan-S, evolved from mEos3.1^19^.

Despite the intensive development of PTFPs, some cellular substructures remain poorly explored at the nanoscale, in particular compartments where oxidative folding takes place, such as the peroxisome, the endoplasmic reticulum, the mitochondrial intermembrane space or the bacterial periplasm. These compartments yet harbour a large number of key macromolecules, involved in e.g. drug uptake, energy production, or oxidative metabolism. The major reason for this gap in the super-resolution field is that fluorescent proteins are generally unable to properly fold and emit light in highly oxidative environments^20^. The development of a PTFP capable of oxidative folding is thus required to facilitate super-resolution imaging of such compartments in living cells.

The periplasm of Gram-negative bacteria, sometimes referred to as the entrance hall of the cell and accounting for 20–40% of its total volume^21^, is involved in important processes such as cell division, environmental signalling and cellular transport^22^. Accordingly, it hosts a variety of proteins involved in antibiotic action (e.g. penicillin binding proteins) and resistance (e.g. beta-lactamases, porins and efflux pumps components)^23^,^24^. Understanding the molecular processes that take place in the periplasm is thus of both fundamental and biomedical interest. Four types of proteins face oxidative folding in the periplasm: secreted proteins, periplasmic proteins, inner membrane proteins and outer membrane proteins. Most outer-membrane, secreted and periplasmic proteins are exported in a post-translational manner, generally in the unfolded state via the Sec secretion pathway, and more rarely in the folded state through the twin arginine translocon (Tat) secretion pathway^25^,^26^. Inner membrane proteins are inserted in a co-translational manner, following recognition of the nascent polypeptide chain emerging from the ribosome by the bacterial signal recognition particle (SRP) and binding of the tripartite complex to the membrane embedded SRP receptor^27^. It was shown that the SRP and Sec system can act cooperatively, to ensure correct insertion of membrane proteins that harbour substantial hydrophilic periplasmic domains^28^. Also, some periplasmic proteins are translocated through the SRP pathway. Although periplasmic GFP fluorescence could be observed after post-folding translocation through Tat^22^, GFP refolding after translocation through Sec has been shown to be problematic inside the periplasm due to undesirable intermolecular disulphide-bridge formation in the oxidative environment^20^. While non-fluorescent, these GFP aggregates showed strong cytotoxicity^29^. In contrast, Superfolder-GFP^20^,^22^,^30^,^31^, a GFP variant engineered for fast maturation and folding kinetics, was shown to enable periplasmic protein localisation studies after Sec-mediated transport, notably when the SRP pathway was employed^30^. Yet Superfolder-GFP is not phototransformable, and is thus unsuited for super-resolution imaging.

Here, we report the rational, structure-based design of two novel superfolding RSFPs, rsFolder and rsFolder2. Both RSFPs are able to fold and efficiently photoswitch in the bacterial periplasm, opening the door to nanoscopic live imaging of macromolecules in such ‘hostile’ compartments. Specifically, we show here that rsFolder2 is suited for RESOLFT imaging of the bacterial periplasm.

## Results and Discussion

### Design and photophysical characterisation of rsFolder

We reasoned that Superfolder-GFP and rsEGFP2, sharing GFP as a common “ancestor”, could be hybridised by pure rational design to provide a reversibly switchable variant capable of efficient folding in the oxidative environment of the *E. coli* periplasm. The four point mutations that allowed evolving EGFP into rsEGFP2 (T65A, Q69L, V163S, A206K)^16^ were thus engineered in Superfolder-GFP, yielding a new PTFP that we named rsFolder. The T65A mutation, known to confer photoswitching capabilities to rsEGFP2^16^, targets the Superfolder-GFP chromophore itself. Two out of the three other mutations target amino-acid positions (163 and 206) that were already modified in Superfolder-GFP relative to the GFP ancestor (see sequence alignment in Supplemental Fig. S1). We chose to retain these rsEGFP2 mutations in rsFolder as it was found that a serine at position 163 is critical to enhance the switching contrast in rsEGFP2^16^, while a lysine at position 206, facing the solvent, confers a strong monomeric character to GFP variants^32^. Thus, when compared to rsEGFP2, rsFolder exhibits six point mutations (S31R, N40Y, S100F, T106N, Y146F and M154T), only one of which occurs in the vicinity of the chromophore (Y146F) (Fig. S1, Fig. S2b).

**Figure 1:**
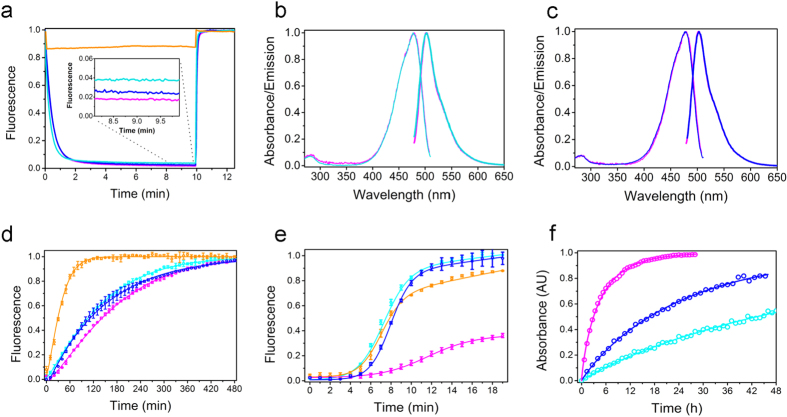
Spectroscopic and biochemical characteristics. Superfolder-GFP (orange), rsEGFP2 (purple), rsFolder (cyan) and rsFolder2 (navy). (**a**) Switching cycle at low illumination intensity (488 nm: 1.9 mW/cm^2^, 405 nm: 2.2 mW/cm^2^). rsFolder switches *off* faster than rsEGFP2 but reaches a higher residual fluorescence in the *off* state (inset). rsFolder2 behaves similarly to rsEGFP2 in terms of switching speed and contrast. Apparent residual switching is solely due its direct fluorescence excitation by the 405-nm laser. (**b,c**) Excitation and emission spectra of rsFolder (b, cyan) and rsFolder2 (c, navy), as compared to those of rsEGFP2 (purple). (**d**) Chromophore maturation kinetics show that the chromophores of rsEGFP2, rsFolder and rsFolder2 (Ala-Tyr-Gly) mature at similar rates while that of Superfolder-GFP (Thr-Tyr-Gly) matures faster. Error bars represent the standard deviation over triplicate measurements. (**e**) Fluorescence recovery kinetics during refolding after chaotropic denaturation by guanidine hydrochloride. The fluorescence signal was monitored continuously at 25 °C. Error bars represent the standard deviation over triplicate measurements. (**f**) Thermal relaxation of the photoswitched *off* states followed at the on-state maximum absorbance for rsEGFP2 (purple), rsFolder (cyan) and rsFolder2 (navy). See Table 1 for values.

The photophysical properties of rsFolder and rsEGFP2 are highly similar (Table 1, Fig. 1 and Fig. S3). As rsEGFP2, rsFolder is an efficient negative photoswitcher, both in purified form (Fig. 1a) and in live cells (Fig. S4). The two proteins display almost identical absorbance and fluorescence spectral properties (Fig. 1b,c). The pKa of the rsFolder chromophore (pKa = 5.5) is slightly lower than that of rsEGFP2 (pKa = 5.9) (Fig. S5, Table 1). Both RSFPs mature slower than Superfolder-GFP (~40 min), with respective half-times of ~2.5 h (rsFolder) and ~3 h (rsEGFP2), (Fig. 1d, Table 1). rsFolder is also monomeric (Fig. S6, Table S1) and refolds as swiftly as Superfolder-GFP (Fig. 1e). Thus, photophysical and biochemical data suggest that rsFolder has inherited both the fast folding properties of Superfolder-GFP and the fast switching properties of rsEGFP2. Its fluorescence emission is in addition maintained over a wide pH range (Fig. S7).

rsFolder yet displays its own peculiarities. First, the thermal stability of its *off* state (~64 hours) is 15 times higher than that of rsEGFP2 (Fig. 1f and Table 1) and, to our knowledge, unprecedented amidst RSFPs. Also, rsFolder exhibits a higher level of residual fluorescence after *off*-switching than rsEGFP2, leading to a reduced switching contrast (Fig. 1a, Fig. S4 and Table 1). This feature arises from the increased *off*-to-*on* quantum yield and the higher extinction coefficient of the *off*-state at 488 nm (Table 1, Fig. S3), is expected to compromise RESOLFT imaging. A possible explanation for these differences is that the two RSFPs photoswitch by different mechanisms. Molecular level insights are required to further substantiate this hypothesis.

### Structural elucidation of rsEGFP2 and rsFolder photoswitching mechanisms

**Figure 2:**
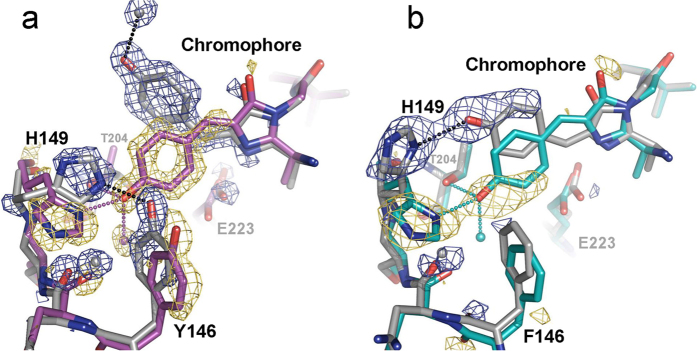
Crystal structures of rsEGFP2 and rsFolder in their *on* and *off* states. Refined models of the chromophores and surrounding residues are shown for rsEGFP2 (**a**) and rsFolder (**b**). Initial *on* states are shown with coloured carbon atoms (purples and cyan for rsEGFP2 and rsFolder, respectively). *Off* states are shown with grey carbons. The F_obs_(*off*)-F_obs_(*on*) difference electron density maps contoured at ±4.5 σ (yellow: negative; blue: positive) highlight atoms exhibiting a notable motion from the fluorescent state (*cis* conformation) to the photoswitched state (*trans* conformation). H-bonds (2.7–2.9 Å) are shown with dashed lines.

With the exception of Dreiklang^33^, all RSFPs of hydrozoan origin have been proposed to undergo *cis-trans* isomerisation of their chromophore upon *off*-switching, by analogy with RSFPs of anthozoan origin^34^. This hypothesis was confirmed recently by structural studies on the rsGreen0.7 variant of rsEGFP^17^. It remains unclear, however, whether rsEGFP2 and rsFolder photoswitch by the same mechanism or not. Inspection of the absorption spectra of rsEGFP2^16^ and rsFolder in their *off* state reveals a broad band centred at ~400 nm. This strongly suggests that in both proteins, the classical rule applies – that is, chromophore protonation couples with isomerisation. To shed light on the switching mechanisms of rsFolder and rsEGFP2, we solved their crystallographic structures in both the *on* and *off* states (Fig. 2 and Table 2). The *on*-to-*off* switching was triggered by illumination of crystals with a fibre-coupled 488-nm laser. Models were refined at 1.45 Å (*on* state) and 1.50 Å (*off* state) resolution for rsEGFP2, and at 1.50 Å (*on* state) and 2.35 Å (*off* state) resolution for rsFolder, respectively. The experimental Fourier difference maps provide evidence that in both RSFPs, *off*-switching indeed results from *cis-trans* isomerisation of the chromophore (Fig. S8). Yet, our structures reveal major differences between the photoswitching mechanisms of rsEGFP2, rsFolder and Dronpa, the anthozoan RSFP archetype^35^. In Dronpa and other negative RSFPs such as mTFP0.7^36^ or IrisFP^13^, a drastic structural reorganisation of the chromophore environment is observed upon switching. The tightly H-bonded Glu144-His193-Glu211 triad in the *cis* conformation is replaced by the Glu144-Arg66-Glu211 triad in the *trans* conformation, with either His193 or Arg66 stabilizing the chromophore by π−stacking and cation-π interactions with the *p*-hydroxybenzylidene moiety, respectively^34^. In rsEGFP2 and rsFolder, on the contrary, no such reorganisation of H-bonding networks is observed and the structural changes are restricted to the near environment of the chromophore phenolate (Fig. 2 and Fig. S8).

In the anthozoan negative RSFPs, it was also noticed that the free energy of the *trans* state is lowered by the ability of Ser142, which maintains a strong H-bond with the hydroxybenzylidene moiety in the *cis* state, to find another H-bonding partner upon chromophore isomerisation^35^,^37^. A similar situation is observed in rsEGFP2, where His149, playing the same role as Ser142 in Dronpa (distance to the chromophore phenolate: 2.7Å), finds Tyr146 as a surrogate H-bonding partner in the *trans* state of the chromophore. This photoswitching mechanism is identical to that recently reported for rsGreen0.7^17^. Of note, in these three RSFPs, the phenolate of the *off*-state chromophore loses all H-bonds to the barrel scaffold, being only H-bonded to a structural water molecule, hitherto absent in the *on*-state structure (Fig. 2a). However, a drastically different scenario is seen in rsFolder, where Tyr146 is replaced by a hydrophobic phenylalanine, one of the mutations inherited from Superfolder-GFP. Consequently, the switching pattern exhibited by rsFolder is unique. In the *on* state, the phenolate oxygen of the *cis* chromophore is H-bonded to Thr204, a water molecule and His149. Upon isomerisation the interactions with the two first partners are lost, while His149 and the phenolate oxygen remain H-bonded, resulting in a *trans* conformation of the chromophore that is a mirror image of the *cis* conformation (Fig. 2b). This highly unusual mechanism results in the *off*-state chromophore of rsFolder remaining tightly attached to the barrel scaffold and likely explains its exceptional thermal stability. The limited number of residues involved in rsFolder *on*-to-*off* switching, and the reduced cascade of H-bond formation and disruption that accompanies it, could result in a higher energy barrier for the *off*-to-*on* transition in rsFolder than in rsEGFP2.

### Design and photophysical characterisation of rsFolder2

The crystallographic structures of rsEGFP2 and rsFolder in their *on* and *off* states provide a clear molecular basis for the observed higher thermal stability of rsFolder’s *off* state. The reduced switching contrast of rsFolder is more difficult to explain in structural terms. The unique symmetry observed between the *on* and *off* states of rsFolder could possibly play a role. Structural data also reveal the central role of the amino-acid at position 146 in differentiating the switching mechanisms of the two RSFPs, and suggest a straightforward strategy to rationally evolve the mechanism of rsFolder into that of rsEGFP2 – that is, replacing its Phe146 by a tyrosine. The F146Y mutant of rsFolder, referred to as rsFolder2, was therefore produced and characterised. As expected, rsFolder2 displays a higher switching contrast (Fig. 1a, Fig. S4) and a reduced thermal stability for its *off* state as compared to rsFolder (Fig. 1f), bringing its photophysical behaviour very close to that of rsEGFP2. Concomitantly, the maturation (Fig. 1d) and refolding kinetics inherited from Superfolder-GFP are essentially preserved in rsFolder2 (Fig. 1d,e). Altogether, these properties (switching contrast, photoresistance, maturation and folding) suggest that rsFolder2 could be a suitable candidate for nanoscale imaging of the bacterial periplasm, using RESOLFT microscopy.

### Expression and RESOLFT imaging in the periplasm

**Figure 3:**
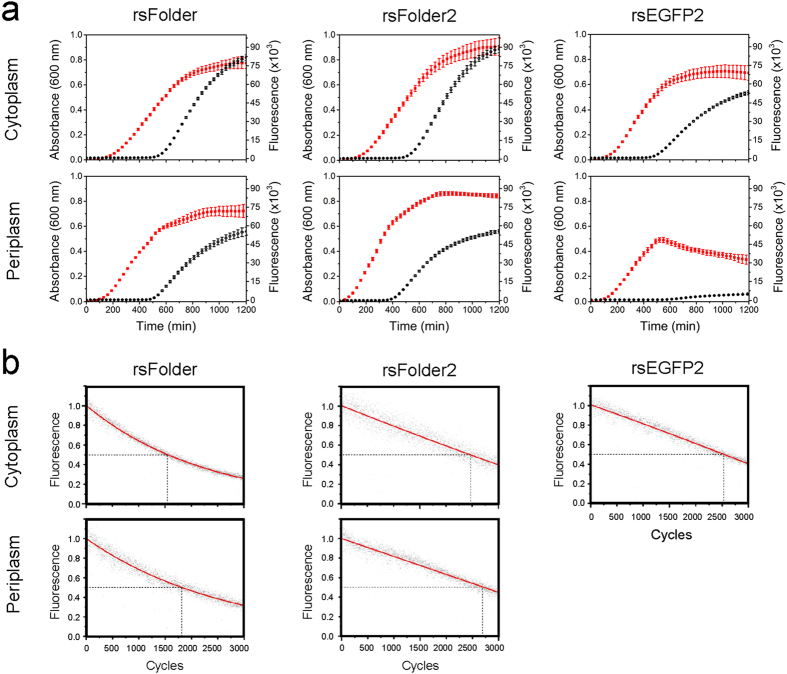
Cytoplasmic and periplasmic expressions and switching fatigue. (**a**) Bacterial growth from a single colony in autoinduction medium and further cytoplasmic and periplasmic expressions of rsFolder, rsFolder2 and rsEGFP2 were monitored in absorbance (red) and in fluorescence (black), respectively, during 20 hours. Error bars represent standard deviations over triplicate measurements. The production of rsEGFP2 targeted to the periplasm corresponds to a brutal stop of bacterial growth that can be explained by cell toxicity induced by periplasmic clogging. (**b**) Switching fatigue measurements of rsFolder and rsFolder2 in comparison to rsEGFP2, expressed in the cytoplasm as well as in the periplasm of living *E. coli* cells. The black dots indicate the maximum *on*-state fluorescence of each switching cycle. The corresponding fitted exponential decay curves are shown in red. The dashed lines illustrate the numbers of cycles the different RSFP samples can achieve before being bleached to half of their initial fluorescence.

Having established that rsFolder and rsFolder2 are both fast-switching and photofatigue-resistant RSFPs and that they refold, *in vitro*, just as well as Superfolder-GFP, we asked whether they could fold and photoswitch in the cellular context, *i.e.* when expressed in the cytosol (pET15-b vector) or the periplasm (pET26-b(+) vector, Sec pathway) of *E. coli* cells. Bacterial cell brightness, here expressed as the ratio between the fluorescence signal (at 505 nm) and the optical density (at 600 nm), informs on the total amount of folded FPs present in living cells. When compared to cytosolic expression, all clones displayed lower bacterial cell brightness in periplasmic expression tests, with the most dramatic effect being observed on cells expressing rsEGFP2 (Fig. S9a). Figure 3 shows that cultures expressing rsEGFP2 into the periplasm die prematurely, presumably owing to the accumulation and deposition of unfolded proteins into toxic aggregates. In contrast, periplasmic expression of rsFolder and rsFolder2 does not affect cell viability (Fig. 3a). The reduced brightness of cells expressing periplasmic rsFolder and rsFolder2 likely originates from the inherently smaller volume of the periplasm, and its regulated overall protein-load. To verify this hypothesis, we separated the periplasmic and cytosolic contents of the cells and evaluated the amount of RSFP present in each compartment by fluorimetry and gel electrophoresis (Fig. S9b). The results support the hypothesis that the amount of protein that can be addressed to the periplasm is indeed limited. Incidentally, they also confirm that the observed fluorescence mostly (~70%) originates from periplasmic RSFPs. Importantly, switching experiments performed on cells grown on solid medium confirm that the switching efficiencies of rsFolder and rsFolder2 are preserved in the cellular context (Fig. 3b and Fig. S4). Data also show that the photofatigue resistance is similar when the RSFPs are addressed to the periplasm.

**Figure 4:**
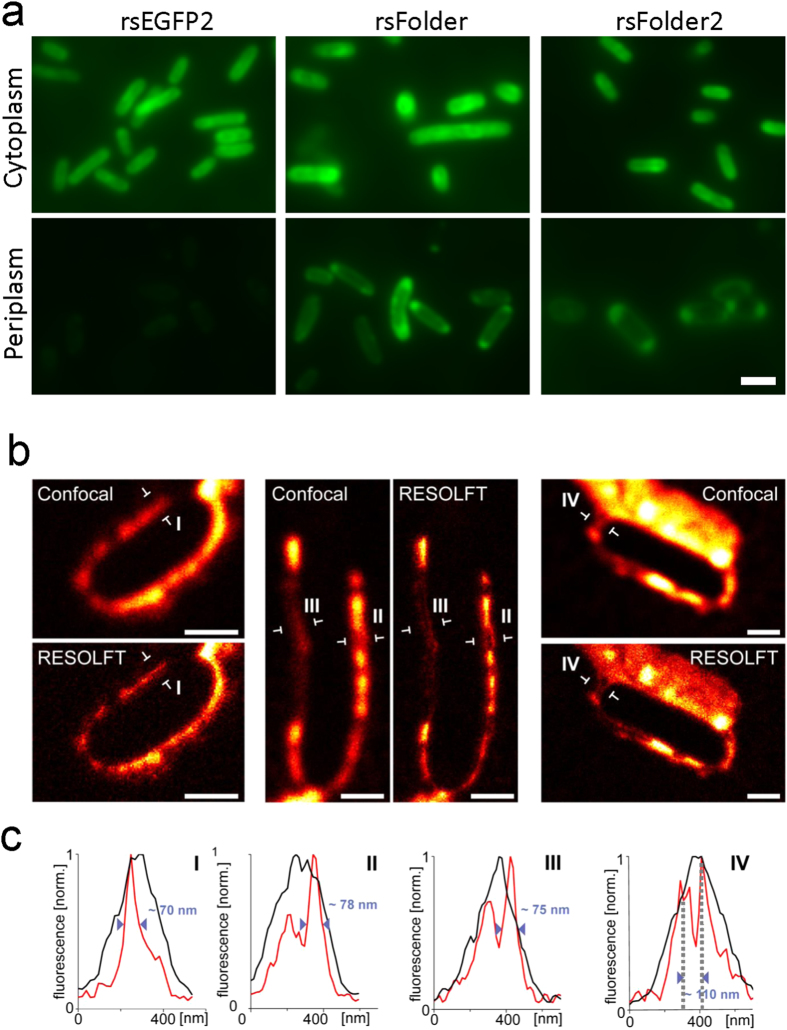
Wide field and RESOLFT Imaging. (**a**) Representative wide field images of fixed *E. coli* bacteria expressing rsEGFP2, rsFolder and rsFolder2 either in the cytoplasm or in the periplasm. All images were obtained using the same illumination conditions and scaled on the same dynamical range. Scalebar: 2 μm. (**b**) RESOLFT imaging of live *E. coli* bacteria expressing rsFolder2 in the periplasm and corresponding diffraction limited confocal images. (**c**) Graphs I-IV illustrate line profiles across the periplasm at the locations indicated by arrows in (**b**). Each profile is an average of five adjacent lines distant by 30 nm. Scalebar: 1 μm

We set to determine whether rsFolder and rsFolder2 could be used for imaging. First, we imaged cells expressing rsEGFP2, rsFolder or rsFolder2 in either the cytosol or the periplasm by wide field imaging (Fig. 4a). Cells expressing the RSFPs in the cytosol show a homogeneous fluorescence distribution, whereas clearly delineated periplasms are visible on images of cells expressing periplasmic rsFolder or rsFolder2. Unsurprisingly, cells expressing periplasmic rsEGFP2 show a hardly detectable signal and no delineation of the periplasm.

We then performed RESOLFT microscopy on *E. coli* cells expressing periplasmic rsFolder2. From measures of the narrowest regions, we derived a resolution of ~70 nm for the periplasm (Fig. 4b, line plot I). Some bacteria showed heterogeneous structures possibly due to the presence of peptidoglycan and associated proteins occupying the periplasmic space (Fig. 4b, line plots II-III). We were also able to distinguish the periplasm of two adjacent bacteria separated by a distance as short as 110 nm (Fig. 4b, line plot IV), demonstrating a significant resolution enhancement compared to diffraction limited imaging. A similar resolution (80 nm) was obtained in controls where a fusion of rsFolder2 with keratin18 was expressed in the cytosol of HeLa cells to benchmark its RESOLFT performance (Fig. S10)^15^,^16^. The RESOLFT micrographs show that the thickness of the periplasm widely varies among cells (Fig. 4b). We correlate this observation with electron micrographs, which reveal that the periplasm is generally larger in isolated cells (up to hundreds of nm) than in colony cells (few tens of nm) (Fig S11a). The heterogeneous nature of the periplasm, and the improvement in resolution allowed by rsFolder2, are further evidenced by the visualisation of what could be outer-membrane budding events and vesicle formation in the RESOLFT images (indicated by arrows in Fig. S11b,c)^38^. Transmission electron micrographs reveal the same features (Fig. S11b). Thus, our results altogether open the door to the live fluorescence super-resolution imaging of complex periplasmic and outer-membrane processes hitherto traceable only by electron microscopy.

The rsFolders are the two first RSFPs endowed with oxidative folding capabilities. We have shown that both are able to fold and photoswitch in the bacterial periplasm, and that rsFolder2 can be used to obtain RESOLFT sub-diffraction images. It remains to be determined whether the rsFolders can be used in other prokaryotic or eukaryotic cellular compartments where oxidative-folding takes place, including the endoplasmic reticulum, thylakoid membranes, the mitochondrial intermembrane space and the external medium^39^. Despite its lower brightness, the second-generation mutant rsFolder2 displays improved switching contrast when compared to rsFolder, making it a well-suited candidate for RESOLFT. We anticipate that this variant could also be useful for other techniques such as patterned activation nonlinear SIM^40^ or pcSOFI^6^ for the imaging of periplasmic proteins in living cells. rsFolder is not adapted for RESOLFT imaging but may prove useful for other super-resolution approaches, such as pcSOFI or potential methods taking advantage of its extreme stability in the *off* state. Further fine-tuning of the photophysical properties of the rsFolders’ scaffold can be envisaged, to better adapt it to other super-resolution techniques. For instance, slow-switching and/or red-shifted rsFolder variants could be developed for single-molecule localisation microscopy and/or multicolour imaging. Specific to RESOLFT imaging, rsFolder2 could be evolved to increase the attainable resolution, either rationally or by directed evolution. Whether or not such improvements are at hand will be determined by future research.

## Methods

### Materials and plasmids

*E. coli* DH5α cells were used for cloning and DNA amplification while *E. coli* BL21 (DE3) were used for expression. Unless otherwise stated, all bacterial cultures were grown in LB medium supplemented with 100 μg/ml ampicillin (cytoplasmic rsFolder, rsFolder2 and rsEGFP2) or 30 μg/ml kanamycine (cytoplasmic Superfolder-GFP and periplasmic rsFolder, rsFolder2 and rsEGFP2). Chemicals were purchased from Sigma-Aldrich.

The Superfolder-GFP/pET-30 and rsEGFP2/pQE31 plasmids were kindly provided by Dr. Cécile Morlot and Prof. Stefan Jakobs, respectively. rsFolder was designed based on the structure of Superfolder-GFP, by introducing into the sequence the four key mutations of rsEGFP2 when compared to EGFP (T65A, Q69L, A163S et V206K). The sequence, synthesised by Eurofins MWG Operon, was recursively codon-optimised both for human and *E. coli* expressions and a Kozak sequence was inserted for potential expression in mammalian cells. Most common restriction sites were removed from the resulting coding sequence to facilitate further cloning uses. rsFolder2 was obtained by directed mutagenesis, using the rsFolder template, and is a single-point mutant of rsFolder (F146Y). For cytosolic expression tests, rsEGFP2, rsFolder and rsFolder2 were subcloned in pET-15b (between *Nde*I and *Bam*HI restriction sites). For periplasmic expression tests, the proteins were subcloned in pET-26b(+) using the Gibson assembly method^41^. Plasmid DNA and PCR fragments were purified with a Qiaprep spin miniprep kit (Qiagen) and a Qiaquick PCR purification kit (Qiagen), respectively. The plasmid encoding the keratin18-rsFolder2 fusion protein was generated by replacing the coding sequence of TagRFP by that of rsFolder2 (restriction sites: *Kpn*I and *Not*I) in the commercial pTagRFP-keratin vector (Evrogen, Moscow, Russia).

### Expression, purification and crystallogenesis

Fluorescent proteins fused to an N-terminal polyhistidine tag were expressed in *E. coli* BL21 (DE3) cells. After cell lysis, the fluorescent proteins were purified by Ni-NTA affinity chromatography followed by size exclusion chromatography using a HiLoad 16/600 Superdex 75 column (GE healthcare, Freiburg, Germany). Purified proteins were concentrated by ultrafiltration and equilibrated in buffer solutions (50 mM HEPES pH 7.5). Crystals of rsEGFP2 and rsFolder were obtained by the hanging-drop vapour diffusion method at 20 °C. Briefly, the protein (12 mg/ml for rsEGFP2 and 10 mg/ml for rsFolder) and precipitant solution (0.1 M HEPES pH 8.1, 1.7 M ammonium sulphate for rsEGFP2 and 0.1 M Tris pH 8.5, 20% PEG 3,350 for rsFolder) were mixed 1:1, yielding 2-μl drops that were placed over a 1 ml well containing the precipitant solution. Crystals appeared within 1–7 days.

### Crystallographic characterisation

Prior to data collection, crystals of rsEGFP2 and rsFolder in their *on* states were cryoprotected by a short soak in the mother liquor supplemented with 15% glycerol, followed by flash-cooling in liquid nitrogen. In order to generate the *off* states, crystals were illuminated for ~1 min with a fibre-coupled 488-nm laser after cryoprotection and before flash-cooling in liquid nitrogen. X-ray diffraction data sets were collected at 100K at the European Synchrotron Radiation Facility (ESRF) on beamlines ID23-2^42^ (rsEGFP2) and ID29^43^ (rsFolder), equipped with a PILATUS 2M and a PILATUS 6M detector, respectively. Data were processed, merged and scaled using XDS/XSCALE, and amplitude factors were generated using XDSCONV^44^. Structures were phased by the molecular replacement method using as a starting model the X-ray structure of Superfolder-GFP (PDB ID: 2B3P) and the program PHASER^45^. Model building was performed with *Coot*^46^. Energy minimisation and individual B-factor refinement followed each stage of manual rebuilding. Refinement and map calculations were performed using REFMAC^47^ and PHENIX^48^. Data collection and refinement statistics are given in Table 2. Figures were produced using PyMOL^49^.

### Analytical ultracentrifugation

Sedimentation velocity experiments were performed in a XL-I analytical ultracentrifuge (Beckman Coulter, USA), with a rotor speed of 42,000 rpm, at 20 °C, using an AnTi-50 rotor. Depending on their concentrations, samples of 55, 110 or 420 μl were respectively loaded in 1.5-, 3-, or 12-mm, path length Ti double-sector centrepieces equipped with sapphire windows (Nanolytics GmbH, Germany). The solvent and reference buffers were 50 mM HEPES pH 7.5; 150 mM NaCl. Radial scans at 280, 488 or 395 nm and from interference optics were monitored. Data were processed using standard methods as implemented in the Sedfit software, v14.6e (www.analyticalultracentrifugation.com) for data analysis. Buffer parameters (in particular, the sedimentation coefficient at 20 °C, *s*_20w_) were calculated using the program Sednterp (sednterp.unh.edu), and assuming a density (*ρ*) and a viscosity (*η*) of 1.008 g.ml^−1^ and 1.05 mPa.s, respectively. Sedimentation velocity profiles were analysed both in terms of continuous distribution *c*(*s*) of sedimentation coefficients (*s)*^50^ and in the framework of the non-interacting species model, providing experimental values for *s* and concentration, on the one hand, and the molar mass *M,* on the other. Predicted partial specific volume 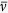 and refractive index increment (∂*n*/∂*c*) were 0.733 ml.g^−1^ and 0.189 for rsFolder, and 0.735 ml.g^−1^ and 0.190 for rsEGFP2, indicating that the two proteins sediment as monomers. Figures were produced using Gussi v1.0.9e (biophysics.swmed.edu/MBR/software.html).

### Spectroscopic characterisation

Absorption spectra were recorded using a Jasco V-630 UV/VIS photospectrometer (Easton, USA). Excitation (λ_em_ = 540 nm) and emission (λ_ex_ = 480 nm) spectra as well as refolding, maturation and expression kinetics were recorded using a Biotek Synergy H4 microplate reader (Winooski, VT, USA). Emission spectra were obtained using excitation at 480 nm while excitation spectra were obtained measuring fluorescence at 540 nm. Molar extinction coefficients were determined using the Ward method^51^. Fluorescence quantum yields were calculated relative to fluorescein (Φ_FL_ = 0.95) and cross-validated between proteins using the method described by Williams *et al.*^52^ pKa values were determined by measuring the anionic absorbance peak of the various FPs as a function of pH, using different buffers [citric acid (pH 3.5), sodium acetate (4.0–5.0), MES (5.5–6.5), HEPES (7.0–8.5), CHES (9.0–9.5)]. Thermal stabilities of the switched-*off* RSFPs were measured by monitoring the absorption as a function of time, every 10 minutes for 1 to 5 days. Photoinduced fluorescence recoveries of switched-*off* FPs were measured at 20 °C with a CCD-based spectrometer (AvaSpec-ULS2048, Avantes, Eerbeek, The Netherlands) coupled with optic fibres to a cuvette holder. Diluted proteins (50 μl) were placed in a 50-μl 3-window cuvette and a square diffuser (Thorlabs, ED1-S50) was put in front of the cuvette to ensure homogeneous laser excitations at 488 nm (1.9 mW/cm^2^) and 405 nm (2.2 mW/cm^2^). Fluorescence emission was measured every 2 s (λ_em_ = 506 ± 4 nm for rsEGFP2, rsFolder and rsFolder2; λ_em_ = 512 ± 4 nm for Superfolder-GFP).

Constant illumination at 488 nm served both to switch *off* the proteins and excite their fluorescence while alternating illumination at 405 nm was used during 150 seconds every 600 seconds to promote *off*-to-*on* recoveries. Evolutions of the fluorescence signal were then analysed with MATLAB (The MathWorks Inc., Natick, Massachusetts, USA) in the framework of the method described in Duan *et al.*^53^, allowing accurate calculation of switching quantum yields.

### Refolding kinetics

All proteins were diluted to 0.1 mg/ml in a denaturation buffer (8 M guanidine hydrochloride, 1 mM DTT, 50 mM HEPES pH 7.5) and heated for 10 minutes at 65 °C. Surprisingly, a treatment by concentrated urea without heating was not sufficient to properly unfold these fluorescent proteins. Denatured proteins were then diluted 10 times in a renaturation buffer (35 mM KCl, 2 mM MgCl_2_, 1 mM DTT, 50 mM Tris pH 7.5, 30% glycerol). For each protein, refolding kinetics were measured by following the fluorescence recovery at 25 °C (λ_em_ = 485 ± 9 nm for all proteins; λ_em_ = 505 ± 9 nm for rsFolder, rsFolder2 and rsEGFP2 and at λ_em_ = 510 ± 9 nm for Superfolder-GFP) every minute during 20 minutes. Data points were fitted along time *t* with the function 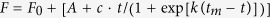 with *k*, the refolding rate; *t*_*m*_, the median refolding time; and the derived lag parameter obtained by 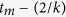. All experiments were performed in triplicate.

### Maturation kinetics

Maturation kinetics were measured using a protocol adapted from Moore *et al.*^54^ Briefly, 50 ml of bacterial cultures were grown in LB medium in 250-ml flasks to an optical density of 0.6 and then transferred into sealed 50-ml tubes. This allowed to create an anaerobic environment that permits protein expression but stops the cell division and chromophores maturation. Proteins were expressed overnight at room temperature after IPTG induction (1 mM). At 4 °C, cells were then harvested by centrifugation, resuspended in 5 ml of lysis buffer (50 mM Tris-HCl, 150 mM NaCl, pH 7.5 mM, 1 tablet of anti-protease cocktail supplemented with DNAseI) and lysed by sonication. After centrifugation, 50 μL of supernatant were added to 200 μL of re-oxygenation buffer (35 mM KCl, 2 mM MgCl_2_, 50 mM Tris-HCl pH 7.5). Fluorescence intensities were recorded at 37 °C every 10 minutes during ~8 hours. All experiments were performed in triplicate.

### Cytoplasmic and periplasmic expressions

To ensure a synchronised start of protein expression and to warrant that no detectable absorbance or fluorescence was present at the beginning of the experiment, bacteria were grown in an auto-inducible medium for these expression tests. Briefly, for each clone, a single bacterial colony was used to inoculate 5 ml of auto induction growth medium^55^ at 25 °C, further distributed into 96-well plates with 100 μl per well. Bacteria were grown using glucose as carbon source until an OD of 0.6 was reached (~8 hours), after what protein expression was initiated by switching to lactose as carbon source, and both OD (600 nm) and fluorescence (505 nm) were monitored as a function of time. Bacterial cell brightness was expressed as the ratio between the fluorescence value and O.D. after 20 h of growth. Cytoplasmic and periplasmic expression levels were further quantified by bacterial fractionation, as previously described^56^. Briefly, induced bacterial cultures were pelleted at 3,000 g for 10 min at 4 °C, resuspended in 0.5 ml TSE buffer (200mM Tris-HCl pH 8, 500 mM sucrose and 1mM EDTA) and incubated on ice for 30 minutes. 0.5 ml of ice cold water was added and incubated for 30 min. Cells were pelleted and the supernatant was collected as the periplasmic fraction. The pellet was resuspended in 5 ml BugBuster protein extraction reagent (Novagen 70921-3) and further incubated for 30 min at room temperature. The lysates were then centrifuged at 16,000 g for 45 min at 4 °C, and the supernatant was collected as the cytoplasmic fraction. Bacterial fractions were loaded on SDS-PAGE 12% acrylamide gels and visualised using Coomassie blue. To quantify folded proteins in each compartment, periplasmic and cytoplasmic fractions were diluted 10 times in HEPES (pH 7.5) and fluorescence intensities were recorded.

### Bacterial photofatigue measurement

Switching fatigue measurements on living *E.coli* colonies were performed by alternate irradiation with laser light of 405 nm (to switch RSFPs *on*) and 488 nm (to switch RSFPs *off*). The light was focus by a 20-fold objective (NA = 0.4) onto a colony, providing power densities of ~0.5 kW/cm^2^ (488 nm) and ~0.1 kW/cm^2^(405 nm). For each RSFP, illumination was kept as short as needed to attain 100% photoswitching. Fluorescence was recorded with a PMT (photomultiplier tube).

### Wide field and RESOLFT microscopy

Bacterial cultures were grown in TB medium supplemented with 1% glycerol to an O.D. of 0.6 and then induced with IPTG overnight at 20 °C. After induction, cells were pelleted, fixed (for wide field imaging) or not (for RESOLFT imaging) with 4% paraformaldehyde, and then washed twice with PBS. A drop of the cell slurry was deposited on glass coverslips previously coated with chitosan (for wide field imaging) or LB agar (for RESOLFT imaging) and mounted onto glass slides.

For mammalian cells experiments, HeLa cells were seeded on cover slides overnight. Transfection was performed using Turbofect (ThermoFisher Scientific) according to the manufacturer’s manual. After 1 day of incubation cells were glued on object slides with picodent twinsil silicone (Picodent, Wipperfürth, Germany), preventing dying, and subsequently imaged.

Wide field imaging experiments were performed on an IX81 Olympus inverted microscope equipped with a 100X oil-immersion objective of 1.49 numerical aperture and an anti-drift NPS nosepiece (Olympus). Samples were illuminated by circularly polarised 488-nm (Spectra-Physics, Santa Clara, USA) 300 μW, 23.5 μm FWHM Gaussian-shaped laser beam.

RESOLFT microscopy was performed using a Quad P microscope (Abberior Instruments, Goettingen, Germany), equipped with a 100X oil immersion objective of 1.4 numerical aperture. RESOLFT images were recorded by applying the following illumination sequence at each scanning position: first rsFolder2 was switched to the *on*-state by illuminating with 405-nm light (30 μs at 2.2 to 2.5 μW). Second a doughnut-shaped beam of 488-nm light (640 to 670 μs at 14 μW) was used to switch the proteins in the periphery of the focal spot into the *off*-state. Third, the fluorescent signal of residual *on*-state proteins at the centre of the spot was recorded at 510 nm following excitation by a 488-nm light (10 to 30 μs at 5 to 7 μW). Before and after *off*-switching with the doughnut-shaped beam, short illumination breaks (up to 60 μs) were introduced into the switching sequence. The scanning step size was set to 30 nm. Detailed information concerning the imaging parameter is provided in table S2.

### Electron microscopy

Transmission electron microscopy was used to visualise *E. coli* BL21 DE3 cells grown in the interstice between LB-agar solid media and an electron microscopy grid^57^,^58^. Briefly, cells were grown overnight to an optical density of ~1 at 600 nm. 20 μL of ten-times diluted cells were deposited on LB-agar media, incubated for 1 h at 37 °C, before adding an electron microscopy grid atop of them. Plates were incubated for 3 additional hours at 37 °C. EM grids were then removed gently, washed 6 times with drop of sterile water and then either imaged directly or after being subjected to negative-staining with 2% (w/v) sodium silicotungstate. Images were taken under low-dose conditions with a CM12 and Tecnai 12 LaB6 electron microscope working at 120 kV and with nominal magnifications of 22,000X and 45,000X using an Orius TM SC1000 CCD camera from Gatan.

## Additional Information

**How to cite this article**: El Khatib, M. *et al.* Rational design of ultrastable and reversibly photoswitchable fluorescent proteins for super-resolution imaging of the bacterial periplasm. *Sci. Rep.* **6**, 18459; doi: 10.1038/srep18459 (2016).

## Supplementary Material

Supplementary Information

## Figures and Tables

**Table 1:** *In-vitro* photophysical and biochemical properties of all proteins presented in this study.

	Superfolder-GFP	rsEGFP2	rsFolder	rsFolder2
Excitation maximum *on* [nm]	487 (488)	479 (478)	477	478
Extinction coefficient (ε) *on*, pH 7.5 [M^−1^.cm^−1^]	54000 (83300)	57150 (61300)	51600	44000
Extinction coefficient (ε) *on*, fully anionic [M^−1^.cm^−1^]	63100	61900	58100	54800
Emission maximum *on* [nm]	510 (510)	503 (503)	503	503
Fluorescence quantum yield on (Φ_fluo_)	0.65 (0.65)	0.35 (0.3)	0.25	0.23
Brightness at pH 7.5 *on* (relative to EGFP)	1.06 (1.64)	0.61 (0.56)	0.39	0.23
*On*-to-*off* switching quantum yield (Φ_off_)	NA	1.65 × 10^−2^	2.1 × 10^−2^	1.98 × 10^−2^
Absorption maximum neutral form [nm]	393	403	403	400
Extinction coefficient (ε) *on* fully neutral [M^−1^.cm^−1^]	36800	27800	29200	25200
Absorption maximum acid/base denatured [nm]	385/450	399/446	399/444	393/444
Chromophore pK_a_, on	5.4	5.9 (5.8)	5.5	5.5
Chromophore maturation rate [10^−4^ s^−1^]	4.2	0.9	1.1	1.1
Refolding rate [10^−2^ s^−1^]	1.5	0.7	1.2	1.6
Absorption maximum *off* [nm]	NA	406 (408)	413	410
Extinction coefficient (ε) *off* [M^−1^.cm^−1^]	NA	22000	27000	23500
Extinction coefficient (ε) *off* at 488 nm [M^−1^.cm^−1^]	NA	60	130	90
*Off*-to-*on* switching quantum yield (Φ_on_)	NA	0.33	0.44	0.28
Switching contrast	NA	~57	~26	~40
*Off*-to-*on* thermal recovery rate [10^−5^ s^−1^]	NA	6.0	0.4	1.1

Values previously reported in different literature sources are shown between parentheses and are extracted from Pédelacq *et al.*, 2006 (Superfolder GFP) and Grotjohann *et al.*, 2012 (rsEGFP2). NA: Not Applicable

**Table 2:** Crystallographic data collection and refinement statistics.

	rsEGFP2 *on*-state	rsEGFP2 *off*-state	rsFolder *on*-state	rsFolder *off*-state
**PDB entry**	5DTX	5DTY	5DTZ	5DU0
Beamline	ESRF/ID23-2	ESRF/ID23-2	ESRF/ID29	ESRF/ID29
Wavelength, Å	0.873	0.873	0.976	0.992
Space group	P2_1_2_1_2_1_	P2_1_2_1_2_1_	C2	C2
*a*, Å	50.99	51.20	142.47	141.29
*b*, Å	62.91	62.81	134.56	134.80
*c*, Å	70.84	70.55	51.72	51.05
α, γ, °	90.0	90.0	90.0	90.0
β, °	90.0	90.0	106.05	105.58
Resolution, Å	47.04-1.45 (1.50-1.45)	46.91-1.5 (1.55-1.5)	95.97-1.50 (1.55-1.50)	95.77-2.35 (2.43-2.35)
*R_sym_, %	5.2 (64.3)	7.5 (60.1)	7.8 (63.7)	10.4 (53.0)
Mean *I*/σ(*I*)	16.1 (2.3)	11.9 (1.2)	9.0 (1.1)	10.1 (2.2)
Completeness, %	99.35 (99.70)	98.13 (91.59)	97.12 (81.62)	98.07 (88.56)
Redundancy	4.3 (4.4)	4.6 (4.1)	4.0 (2.8)	4.0 (4.0)
Unique reflections	40827 (3787)	36769 (3364)	144849 (12182)	37525 (3391)
Wilson B factor, Å^2^	13.76	14.85	20.82	39.94
#R_*work*_/R_*free*_	0.17/0.21	0.18/0.22	0.18/0.20	0.21/0.26
Average B factor, Å^2^	18.9	21.9	25.0	41.3
Rmsd				
* Bond length, Å*	0.014	0.018	0.010	0.007
* Bond angles, °*	1.65	1.76	1.33	1.07
* Favored*	98.0	97.0	99.0	98.0
* Allowed*	2.0	1.8	0.78	1.78
* Outliers*	0.0	1.2	0.22	0.22

Values in parentheses refer to the highest resolution shell.

^*^R_sym_ = ΣjΣh|I_h,j_ – 〈Ih〉|/ΣjΣh I_h,j_

^#^R_*work*_ = Σh|F_obs_ – F_cal_|/ΣhF_obs,_ R_*free*_ is calculated with a small fraction (5%) of reflections chosen to be part of a test group.
