# Supplementary material for: Rational design of ultrastable and reversibly photoswitchable fluorescent proteins for super-resolution imaging of the bacterial periplasm
Source: Sci Rep. 2016 Jan 6;6:18459. doi: 10.1038/srep18459 (PMC4702087; doi:10.1038/srep18459)
Supplement: Supplementary Information [file srep18459-s1.pdf]

Rational design of ultrastable and reversibly photoswitchable fluorescent proteins for super-resolution imaging of the bacterial periplasm.

Supplementary material

Mariam El Khatib[1,2,3], Alexandre Martins[1,2,3], Dominique Bourgeois[1,2,3], Jacques-Philippe Colletier[*,1,2,3] and Virgile Adam[*,1,2,3]

[1]*Univ. Grenoble Alpes, IBS, F-38044 Grenoble, France,* [2]*CNRS, IBS, F-38044 Grenoble, France,* [3]*CEA, IBS, F-38044 Grenoble, France.*

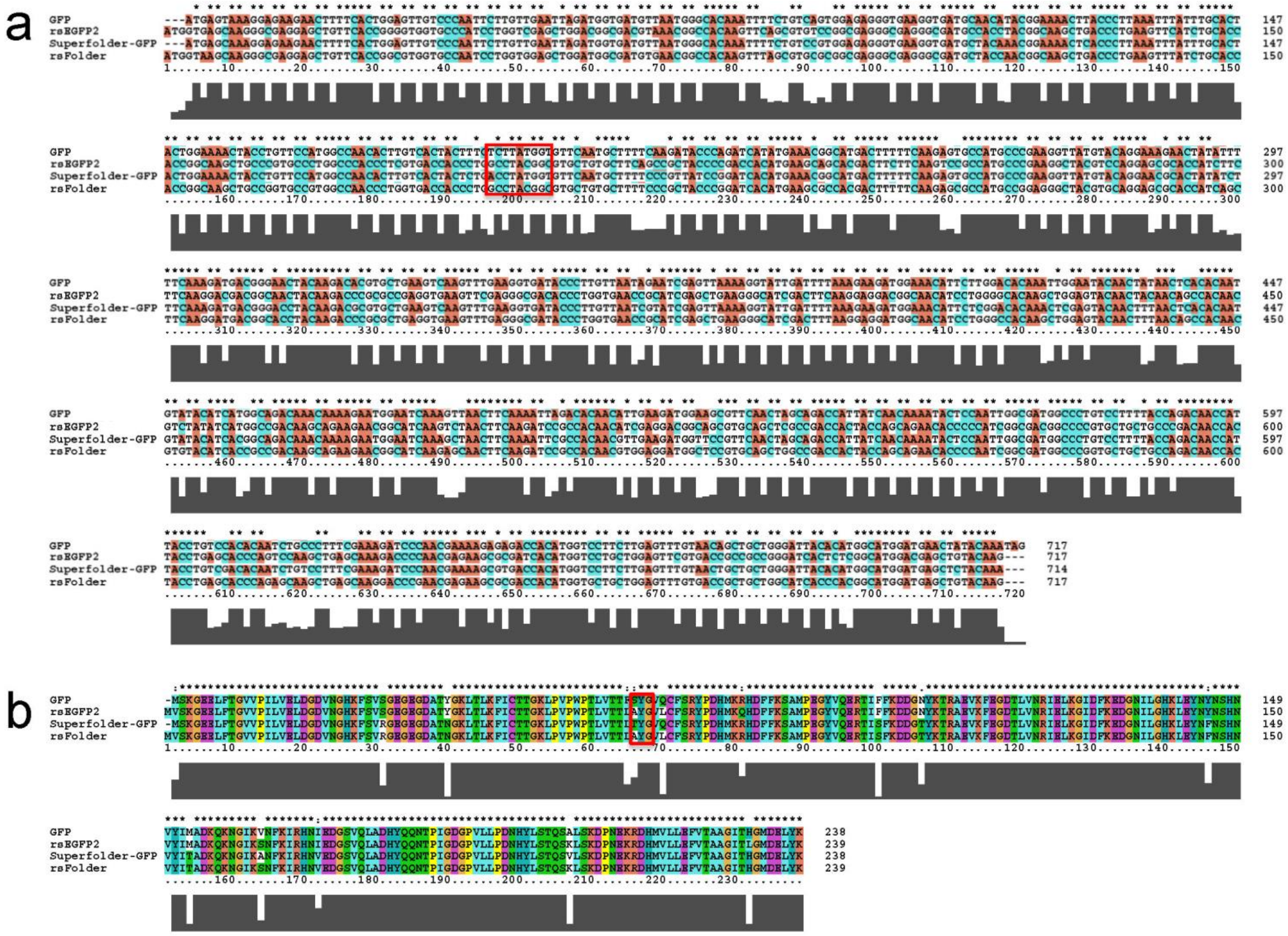

Figure S1. Sequence alignments. Nucleic acid (a) and amino-acid (b) alignments are shown for rsEGFP2, Superfolder-GFP and rsFolder, relatively to GFP. Sequences corresponding to the chromophores are red-squared. Colour-coded by default by the ClustalX alignment software.

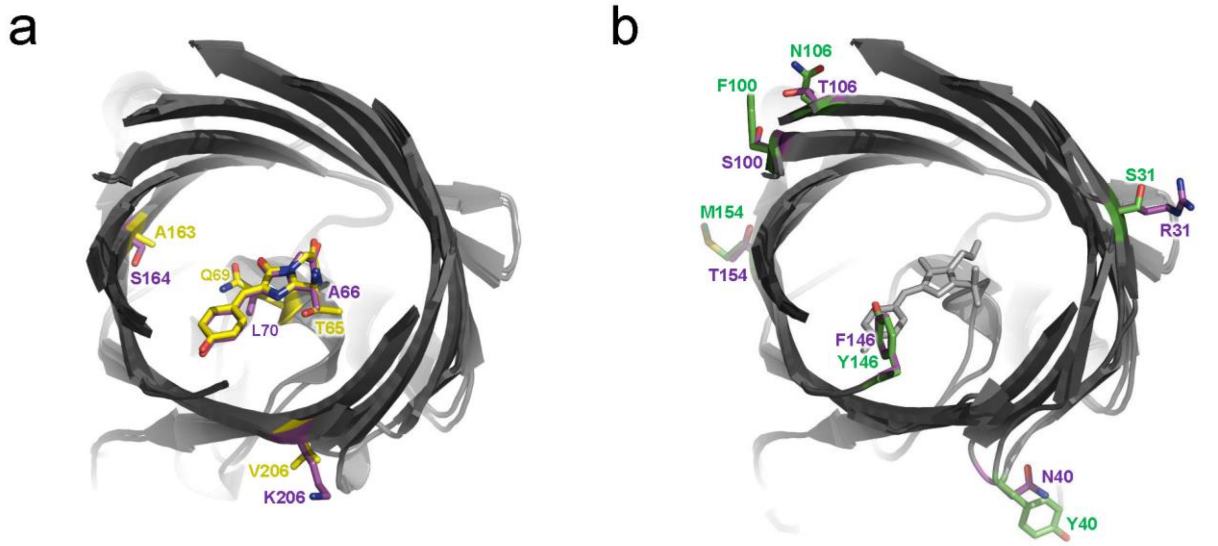

Figure S2. Representation of mutations of rsFolder as compared to rsEGFP2 and Superfolder-GFP. rsFolder was engineered from the architecture of Superfolder-GFP. (a) Four key residues in Superfolder-GFP (yellow) were mutated (purple). (b) The resulting rsFolder contains six aminoacid substitutions as compared to rsEGFP2 (green).

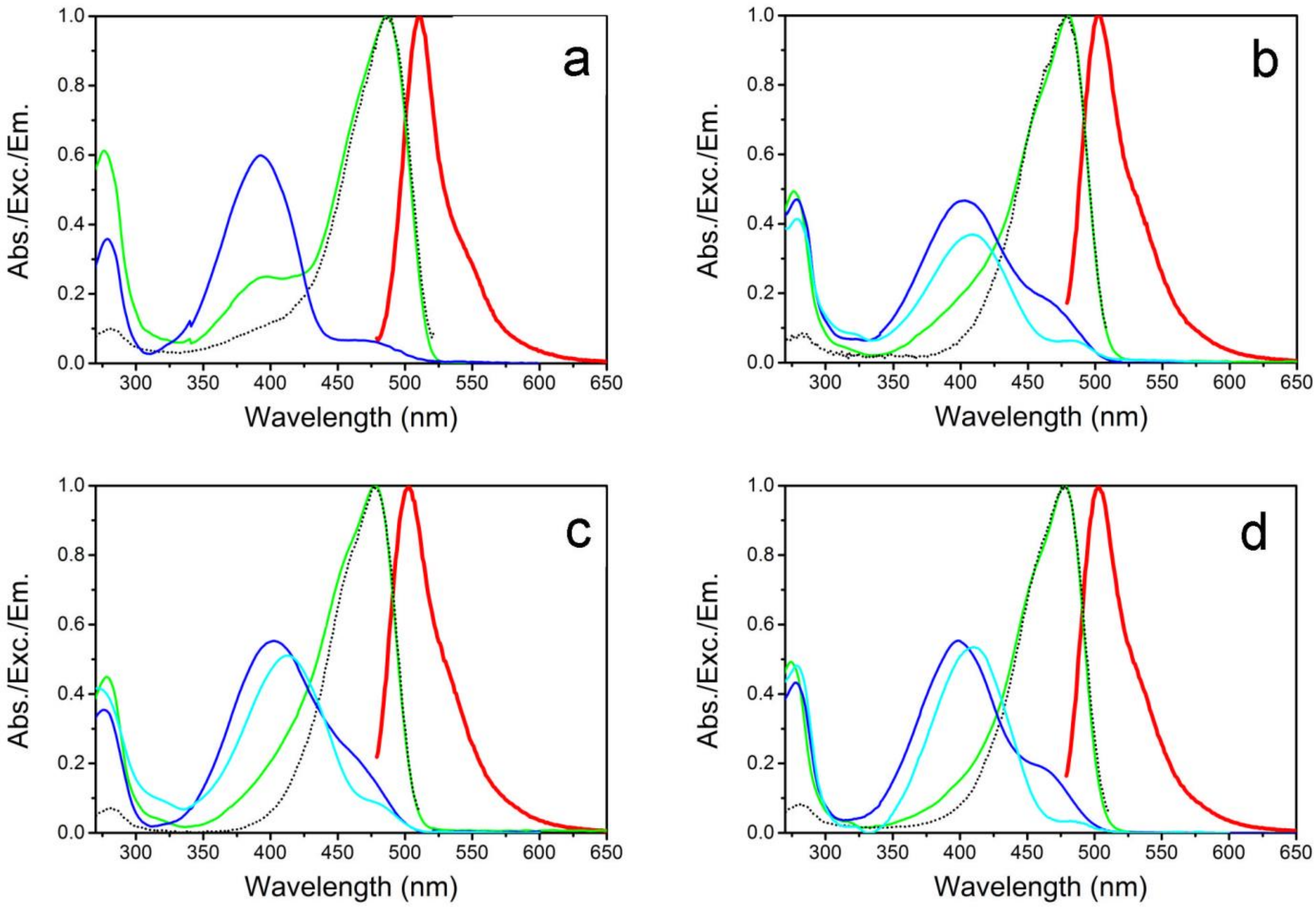

Figure S3. Spectroscopic characterization. Superfolder-GFP (a), rsEGFP2 (b), rsFolder (c) and rsFolder2 (d). Absorption spectra at pH 7.5 are shown for the *on* state (green) and photoswitched *off* state (cyan). Excitation spectra of the *on* state are shown as dotted black lines and corresponding emission spectra as plain red lines. Absorption spectra of the neutral state for Superfolder-GFP (pH 3.4) rsEGFP2 (pH 5.0), rsFolder (pH 4.5) and rsFolder2 (pH 4.0) are shown in blue.

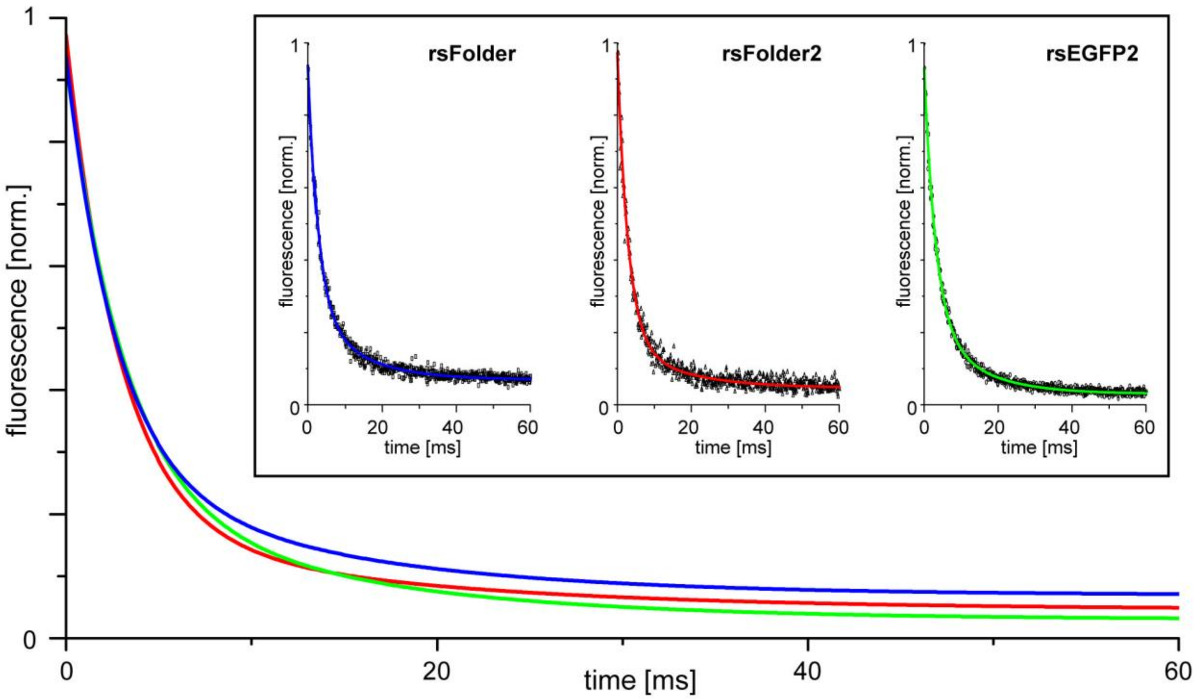

Figure S4. *Off*-switching kinetics of rsFolder, rsFolder2 and rsEGFP2 recorded on living *E. coli* samples. Comparison of fitted exponential decay curves representing the *off*-switching kinetics of rsFolder (blue), rsFolder2 (red) and rsEGFP2 (green). The inset shows raw data for each protein and the corresponding fitted curves. *Off*-switching was performed using a 488-nm laser light on *E.coli* colonies grown on agar plates. The light was focused with a 20X objective lens (NA = 0.4) to an intensity of ~ 0.5 kW/cm² and fluorescence light was recorded by a PMT (photomultiplier tube).

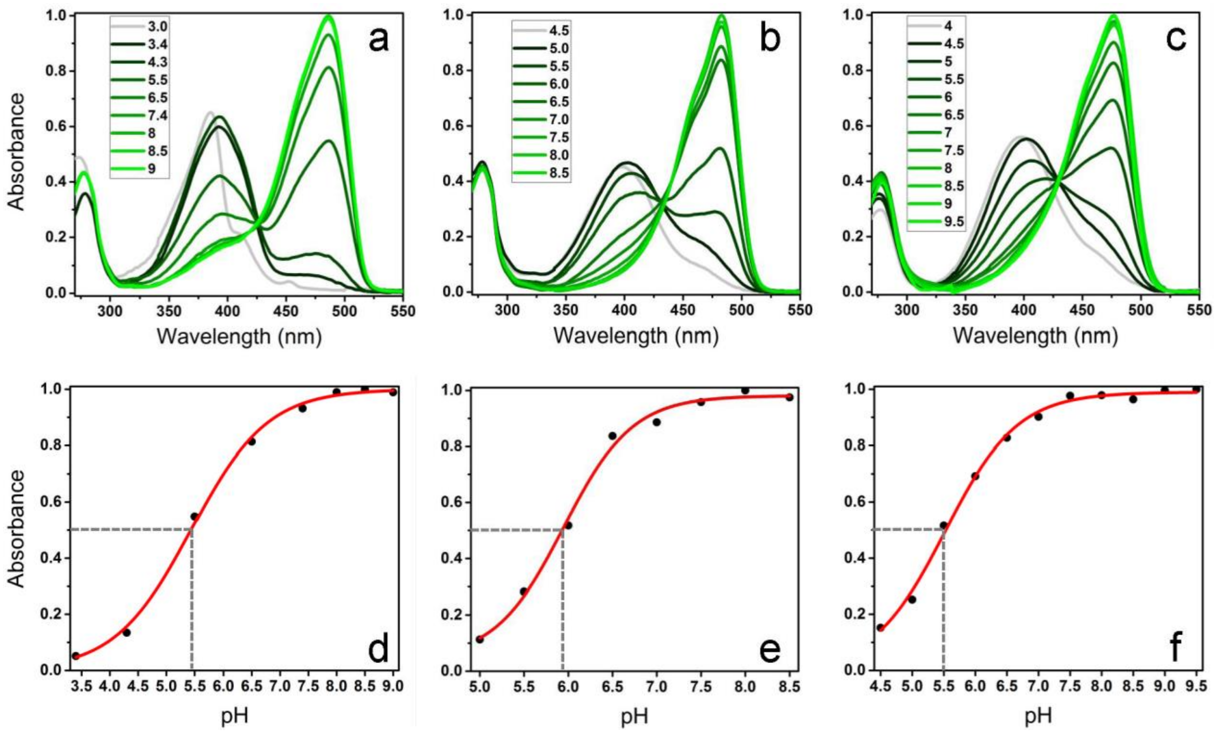

Figure S5. pH dependency of Superfolder-GFP (a,d), rsEGFP2 (b,e) and rsFolder (c,f) absorbance. (a-c) absorbance spectra colour-coded from black to green as a function of increasing pH. The gray spectrum represents the pH value at which denaturation starts to be spectroscopically detected. (d-f) Data points (black dots) corresponding to the absorbance of the anionic peak, fitted by a Henderson-Hasselbalch equation. Gray dotted lines represent the apparent pKa.

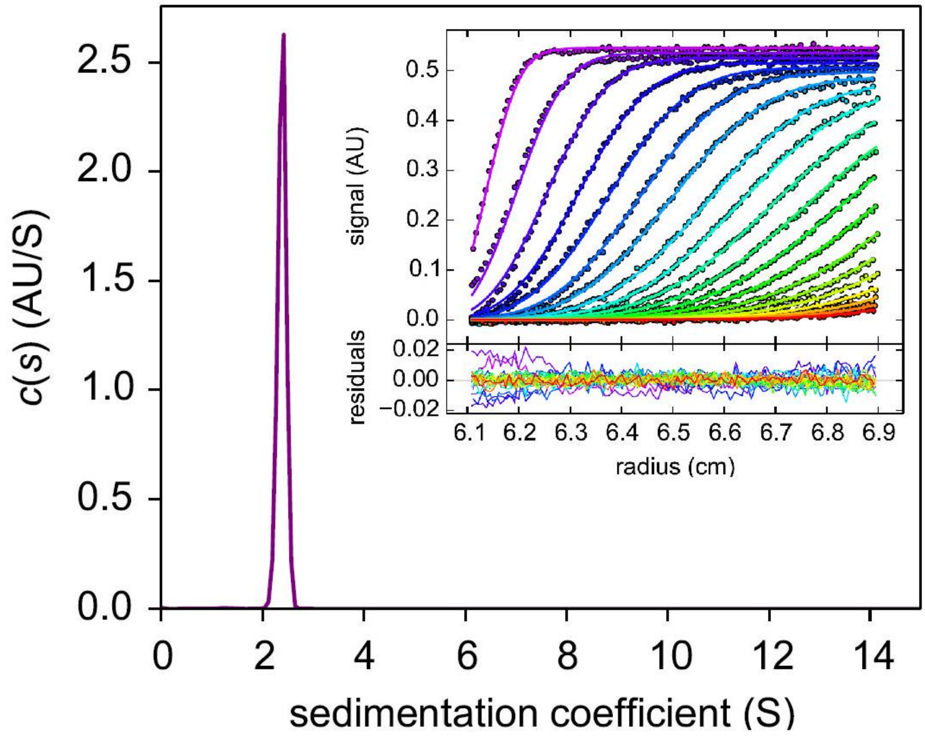

Figure S6. Sedimentation velocity of rsFolder and rsEGFP2. Analytical ultracentrifugation data show that both rsEGFP2 and rsFolder are monomeric. Sedimentation velocity experiments were performed on rsFolder at 0.8, 3.3, 19 and 41 mg mL^{-1}, to probe its association state in solution. rsEGFP2 was investigated at 2 and 22 mg mL^{-1} for comparison. The two proteins sediment with a contribution at ≈ 2.5 S as demonstrated by *c*(*s*) analysis. (Inset) Superposition of experimental (colour-scattered as a function of centrifugation times from purple to dark red) and fitted sedimentation velocity profiles measured at 395 nm for rsFolder at 19.4 mg mL^{-1} (top), and residuals (bottom).

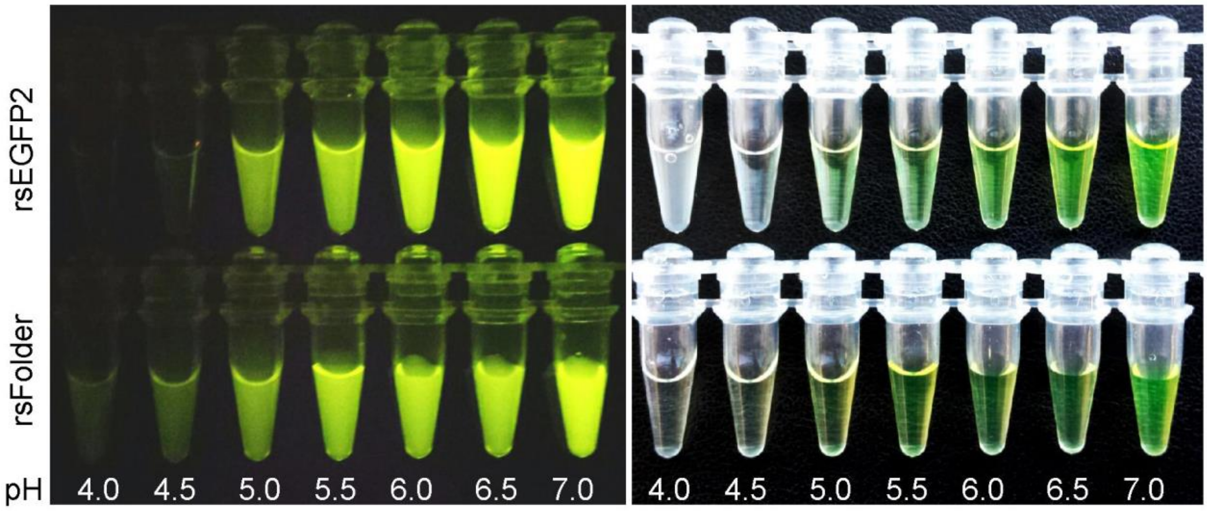

Figure S7. pH-dependency of the fluorescence of rsEGFP2 and rsFolder. For pH values superior to 5.0, rsEGFP2 and rsFolder show a similar fluorescence gradient with varying pH. At pH lower than 5.0, however, rsEGFP2 starts to precipitate as assessed by the cloudy aspect of the protein solution observed under white light (right panel). This precipitation results in no fluorescence at low pH when rsFolder is still properly folded and fluorescent.

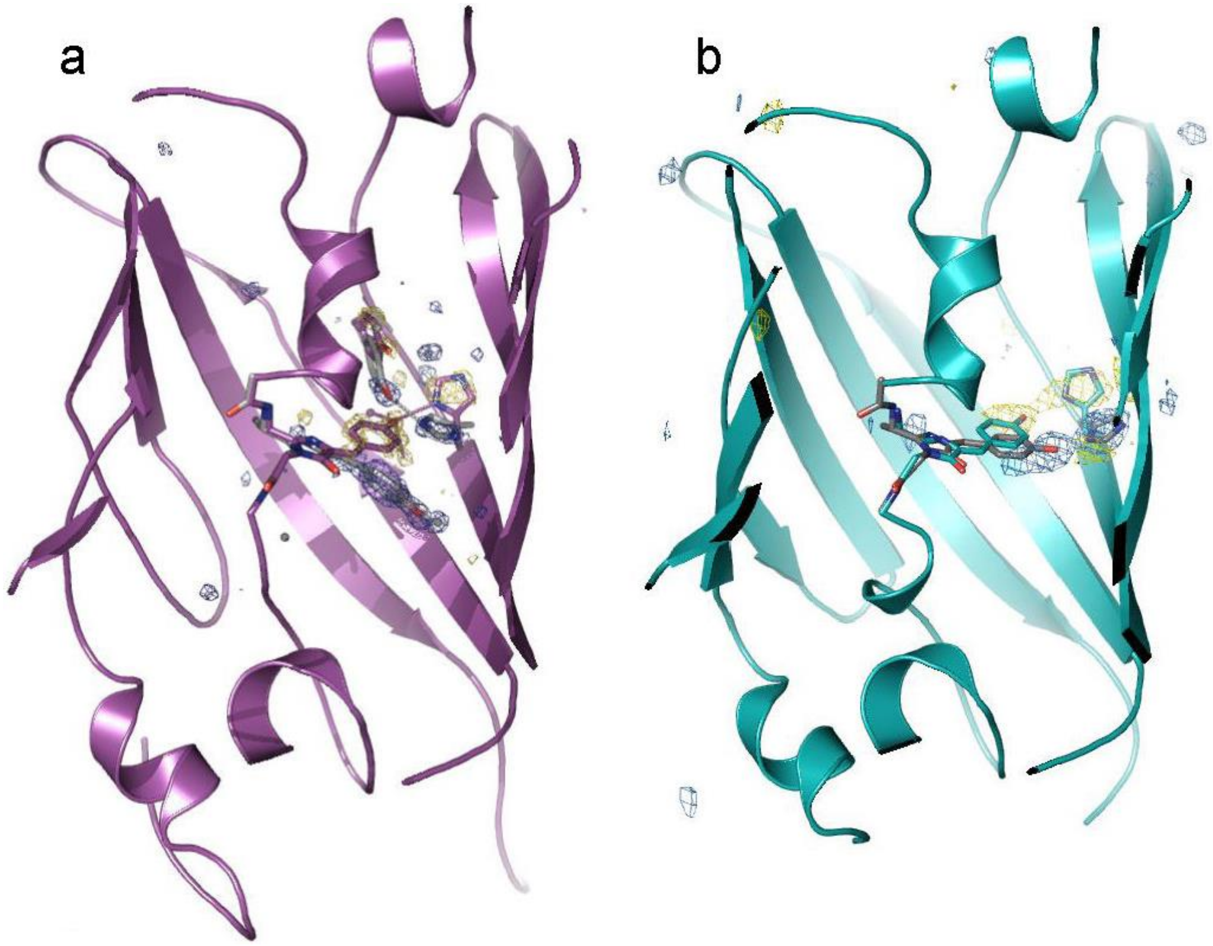

Figure S8. Overall view of structural changes upon *cis*/*trans* isomerization. Both in rsEGFP2 (a) and rsFolder (b) all the structural modifications are limited to the chromophore and its direct environment as depicted by electron density difference maps (± 5 σ) extended to the whole β-barrel.

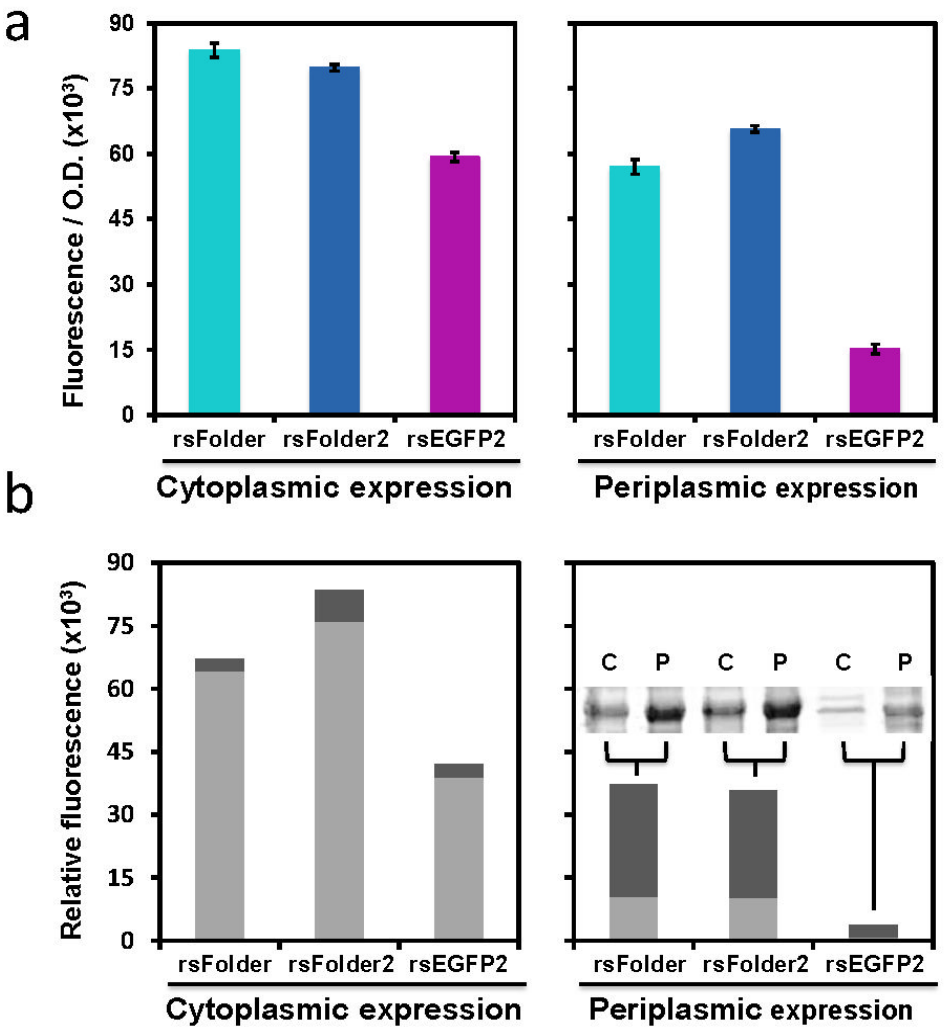

Figure S9. Quantification of cytoplasmic and periplasmic expression of rsEGFP2, rsFolder and rsFolder2. (a) Bacterial cell brightness expressed as the ratio between the fluorescence signal at 510 nm and the optical density at 600 nm at the beginning of the stationary phase of the bacterial growth that was monitored as a function of time on *E. coli* cells grown at 37°C in liquid media and expressing rsEGFP2, rsFolder or rsFolder2. (b) Cytoplasmic and periplasmic contents of *E. coli* cells expressing rsEGFP2, rsFolder and rsFolder2 were isolated. In spite of different intensities, fluorescence measurements demonstrate similar signal ratios between cytoplasmic (light gray) and periplasmic (dark gray) for all three FPs either targeted to the cytoplasm (pET15-b vector) or to the periplasm (pET26-b(+) vector). As expected, the fluorescence signal originates mostly from periplasmic fractions when pET26-b(+) is used. Unlike the rsFolders, rsEGFP2 is almost non-fluorescent when secreted to this compartment although it is efficiently translocated as assessed by SDS-PAGE. In the case of periplasmic secretion, the fluorescence detected in cytoplasmic fractions could originate from fast folding in the cytoplasm of a fraction of the rsFolder and rsFolder2 proteins, which would prevent their secretion. However, the detection of residual periplasmic fluorescence when the RSFPs are targeted to the cytosol points at some cross contaminations between the cytoplasmic and periplasmic contents during cell fractionation.

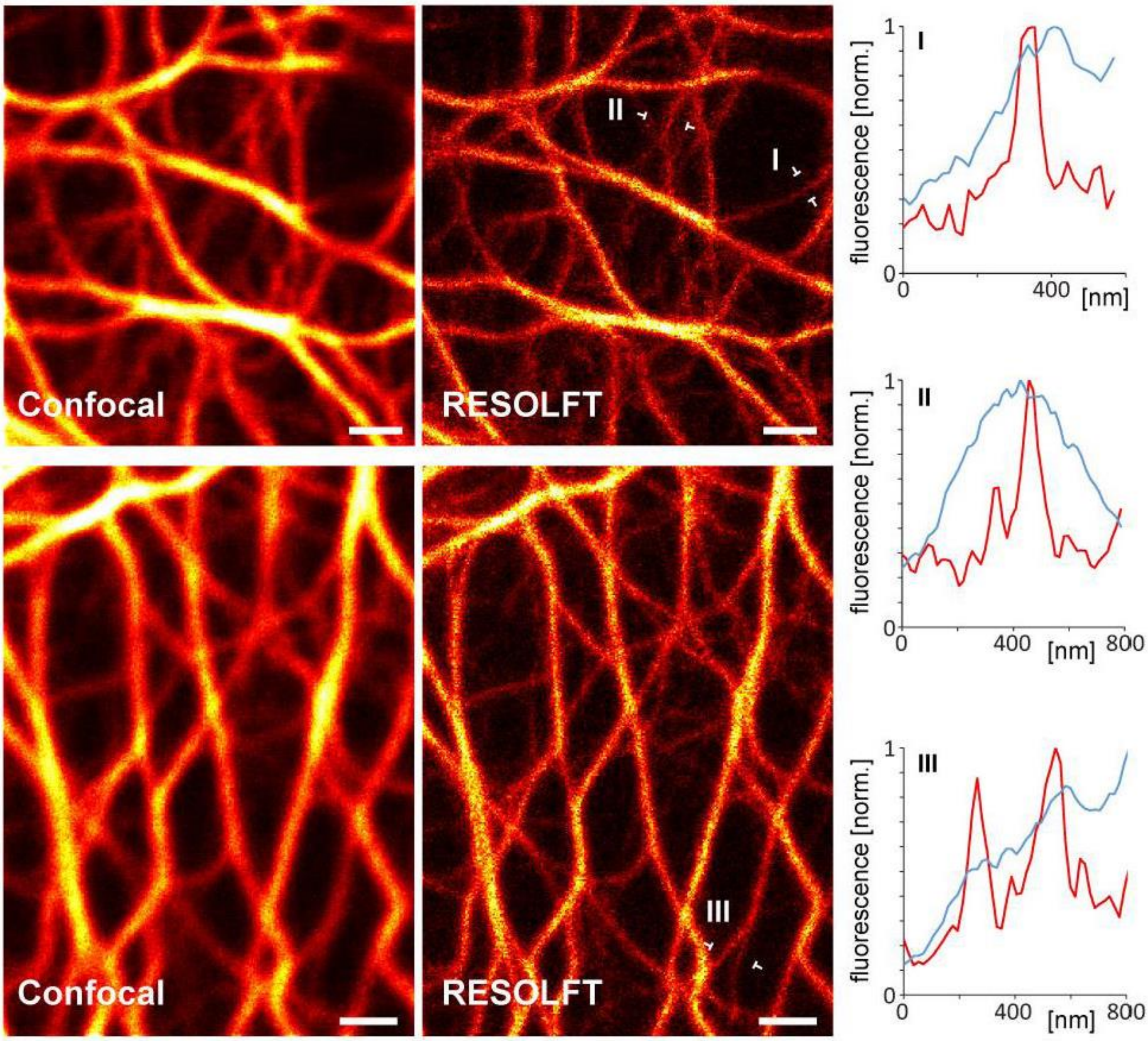

Figure S10. RESOLFT microscopy of living HeLa cells expressing a keratin18-rsFolder2 fusion protein. Two RESOLFT images of keratin18 structures in HeLa cells labeled by rsFolder2, and their corresponding confocal images are shown. Graphs I – III illustrate line profiles across single keratin18 filaments at the locations indicated by arrows. Each of these profiles are a sum of 5 adjacent line profiles distant by 30 nm. RESOLFT images show filaments with diameters < 80 nm. Scale bar 1 µm.

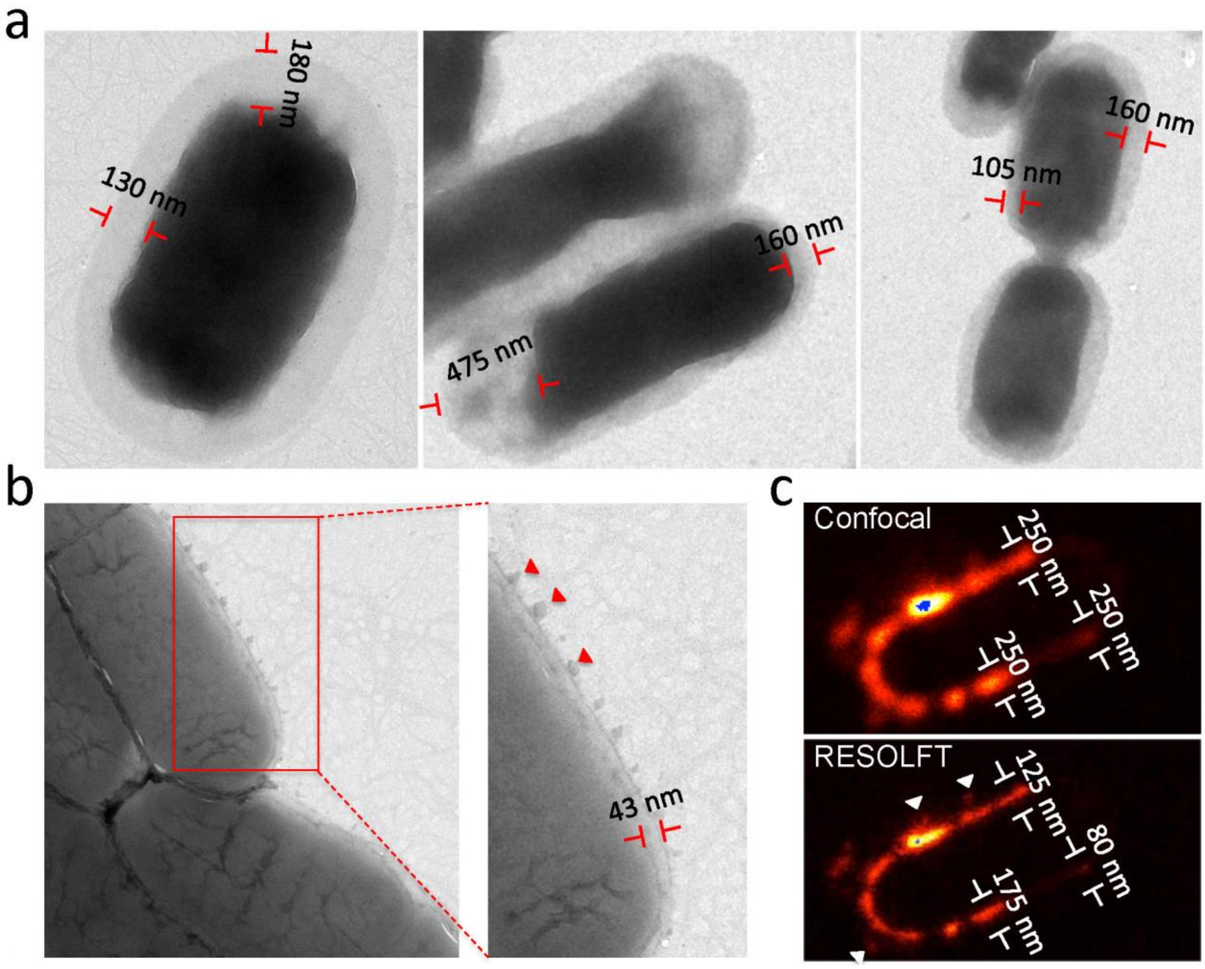

Figure S11. Transmission electron micrographs of *E.coli* compared to confocal and RESOLFT microscopy. (a-b) Bacteria were grown in the interstice between an electron microscopy grid and LB-agar medium for 4h at 37°C. (a) The grid was imaged without negative staining and showing variable periplasmic width (100 nm – 475 nm) of isolated cells. (b) The grid was imaged with negative staining and showing outer membrane budding event (red arrows) and a smaller periplasmic space between colony cells. (c) Diffraction limited confocal imaging and the corresponding RESOLFT imaging of bacteria expressing rsFolder2 in the periplasm showing similarly an uneven periplasmic width and some budding outer membrane vesicles (white arrows).

Table S1. Results obtained in analytical ultracentrifugation

	C (mg/ml)	S_{20W} (S)	M (kDa)
**rsFolder**	40.8	2.27	
	19.4	2.57	
	3.3	2.67	26.8
	0.8	2.68	26.8
	0.0	2.69	
**rsEGFP2**	21.8	2.55	
	1.7	2.77	
	0.0	2.80	

Table S2. Imaging parameters applied for RESOLFT microscopy

**RESOLFT figure**	***On*-switching beam (405 nm)**		**Pause**	**Depletion beam (488 nm)**		**Pause**	**Readout beam (488 nm)**	
	Power (μW)	Illumin. time (μs)	μs	Power (μW)	Illumin. time (μs)	μs	Power (μW)	Illumin. time (μs)
**Fig. 4b left & S11c**	2.5	30	60	14	650	20	5	10
**Fig. 4b mid. & right**	2.5	30	60	14	640	20	5	10
**Fig. S10 top**	2.2	30	10	14	670	0	5	30
**Fig. S10 bottom**	2.5	30	60	14	650	20	7	30

All light powers were measured at the back focal plane of the objective lens
